# In Situ Synthesis of Silver Nanoparticles on the Polyelectrolyte-Coated Sericin/PVA Film for Enhanced Antibacterial Application

**DOI:** 10.3390/ma10080967

**Published:** 2017-08-18

**Authors:** Rui Cai, Gang Tao, Huawei He, Pengchao Guo, Meirong Yang, Chaoxiang Ding, Hua Zuo, Lingyan Wang, Ping Zhao, Yejing Wang

**Affiliations:** 1 College of Biotechnology, Southwest University, Beibei, Chongqing 400715, China; cairui0330@email.swu.edu.cn (R.C.); wly20080108@swu.edu.cn (L.W.); 2 State Key Laboratory of Silkworm Genome Biology, Southwest University, Beibei, Chongqing 400715, China; taogang@email.swu.edu.cn (G.T.); guopc@swu.edu.cn (P.G.); yangmeirong@email.swu.edu.cn (M.Y.); 13060221549@163.com (C.D.); zhaop@swu.edu.cn (P.Z.); 3 Chongqing Engineering and Technology Research Center for Novel Silk Materials, Southwest University, Beibei, Chongqing 400715, China; 4 College of Pharmaceutical Sciences, Southwest University, Beibei, Chongqing 400715, China; zuohua@swu.edu.cn

**Keywords:** sericin, poly(vinyl alcohol), polyelectrolyte multilayers, AgNPs, antibacterial activity

## Abstract

To develop silk sericin (SS) as a potential antibacterial biomaterial, a novel composite of polyelectrolyte multilayers (PEMs) coated sericin/poly(vinyl alcohol) (SS/PVA) film modified with silver nanoparticles (AgNPs) has been developed using a layer-by-layer assembly technique and ultraviolet-assisted AgNPs synthesis method. Ag ions were enriched by PEMs via the electrostatic attraction between Ag ions and PEMs, and then reduced to AgNPs in situ with the assistance of ultraviolet irradiation. PEMs facilitated the high-density growth of AgNPs and protected the synthesized AgNPs due to the formation of a 3D matrix, and thus endowed SS/PVA film with highly effective and durable antibacterial activity. Scanning electron microscopy, energy dispersive spectroscopy, X-ray diffractometry, Fourier transfer infrared spectroscopy, water contact angle, mechanical property and thermogravimetric analysis were applied to characterize SS/PVA, PEMs-SS/PVA and AgNPs-PEMs-SS/PVA films, respectively. AgNPs-PEMs-SS/PVA film has exhibited good mechanical performance, hydrophilicity, water absorption capability as well as excellent and durable antibacterial activity against *Escherichia coli*, *Staphylococcus aureus* and *Pseudomonas aeruginosa* and good stability and degradability. This study has developed a simple method to design and prepare AgNPs-PEMs-SS/PVA film for potential antibacterial application.

## 1. Introduction

Silk sericin (SS) is a globular protein produced by *Bombyx mori* (silkworm) [1], which contains 18 amino acids including the essential amino acids required by the human body [2]. It is about 20–30% of the total weight of silk cocoon. The main role of sericin is to envelop the fibroin [3]. As a natural protein, silk sericin is biocompatible and biodegradable. Since sericin has a variety of additional properties like gelling ability, water-holding capacity and skin adhesion [4], it has been widely applied in the biomedical field. Moreover, silk sericin could promote the adhesion and proliferation of human skin and accelerate burn or scald wound healing [5,6,7]. Therefore, it is regarded as a good candidate for wound dressing material. However, sericin itself is frangible and has a poor mechanical performance, which is not favorable for its application in wound healing. In our previous study, we have prepared sericin/poly(vinyl alcohol) (SS/PVA) blend film to improve the mechanical property of sericin [8]. Bacterial infection is a huge challenge for wound healing. To resolve this problem, it is necessary to functionalize the SS/PVA film with good antibacterial activity.

Antimicrobial surface coating or modification has attracted more and more attention in biomedical and industrial fields [9,10,11,12]. With the rapid development of nanotechnology, a lot of novel nanomaterials have been created with exciting chemical and physical properties [13,14]. As an essential metal nanometer material, silver nanoparticles (AgNPs) have a broad range of applications, from electronics [15] and catalysis [16] to infection prevention [17] and medical diagnosis [18]. It is an effective broad-spectrum antimicrobial agent [19,20,21] and rarely leads to the development of resistant microbes [22]. Compared with other antibacterial agents, AgNP has low cytotoxicity to human cells [23,24]. In addition, AgNP is an effective anti-inflammatory agent and is used to promote wound healing [25]. To date, a number of chemical and physical methods have been developed to synthesize AgNPs [26,27,28,29]. In the traditional methods, toxic chemical reagents and expensive instruments such as ultrasound microwave [30] and γ-radiation [31] are required for AgNPs synthesis. Hence, it is necessary to develop a green and economical method to prepare AgNPs. Ultraviolet (UV)-assisted AgNPs synthesis is one of the most simple and environmentally friendly methods [32]. Studies have shown that AgNPs are commonly immobilized on the material’s surface [33,34]. In this case, Ag^+^ binding sites are limited on the surface of material, so only limited AgNPs could be synthesized. In addition, the synthesized AgNPs are unstable due to the weak van der Waals interactions, which may reduce its bactericidal efficiency. Polymers such as poly(vinyl pyrrolidone), poly(acrylic acid) and polyamide could provide more binding sites for Ag^+^ [35,36]. Layer-by-layer self-assembly is a simple technique to construct polyelectrolyte multilayers (PEMs) [37,38]. It is based on the sequential adsorption of oppositely charged components, in which two oppositely charged polymers are deposited on the surface of material by means of electrostatic attraction [39,40,41,42]. PEMs could provide more Ag^+^ binding sites and create a three-dimensional (3D) space for the growth of AgNPs, thus improving the loading of AgNPs and protecting the entrapped AgNPs.

In this study, we have established a poly(acrylic acid) (PAA)/poly(dimethyl diallyl ammonium chloride) (PDDA)/PAA multilayered structure on the surface of SS/PVA film, which acted as a three-dimensional matrix to allow the high density synthesis of AgNPs *in situ via* the UV-assisted AgNPs synthesis and protect the synthesized AgNPs from oxidation and falling off. Scanning electron microscopy (SEM), energy dispersive spectroscopy (EDS), X-ray diffractometry (XRD), Fourier transfer infrared spectroscopy (FT-IR), water contact angle, mechanical property and thermogravimetric analysis (TGA) were performed to characterize the prepared films. Antimicrobial assays were conducted to investigate the antimicrobial performance of AgNPs-PEMs-SS/PVA film against *Escherichia coli* (*E. coli*), *Staphylococcus aureus* (*S. aureus*) and *Pseudomonas aeruginosa* (*P. aeruginosa*). This novel composite film has exhibited great potential in biomedical applications for its excellent performance and durable antimicrobial activity.

## 2. Results and Discussion

### 2.1. Preparation of PEMs-SS/PVA Film

**Figure 1:**
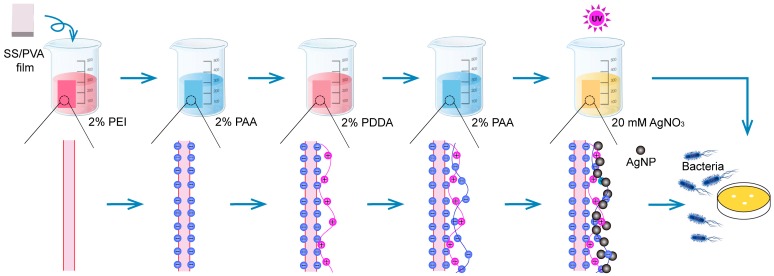
Schematic diagram of the preparation and antibacterial characterization of AgNPs-PEMs-SS/PVA film.

Ito et al. have prepared a highly flexible, strong adhesion, and transferrable AgNPs-loaded polymer nanosheet using the layer-by-layer assembly technique [43]. Here, poly (ethyleneimine) (PEI) is a cationic polymer and could adhere on SS/PVA film surface via the electric interaction between PEI and the negatively charged groups exposed on the surface of SS/PVA film. PAA and PDDA are two weak polyelectrolytes with negative and positive charges, respectively, which are used to construct a stable PEM on the surface of SS/PVA film for the adsorption of Ag^+^. The alternative treatment with PAA and PDDA resulted in the formation of a three-dimensional matrix on the surface of SS/PVA blend film. AgNPs were synthesized in situ on the film surface by UV irradiation. PEMs provided a three-dimensional space for the high-density growth of AgNPs and protected the synthesized AgNPs, thus endowing SS/PVA film with highly effective and durable antibacterial activity. The preparation and antibacterial characterization of AgNPs-PEMs-SS/PVA film were described in Figure 1.

### 2.2. SEM, EDS and XRD Analysis

**Figure 2:**
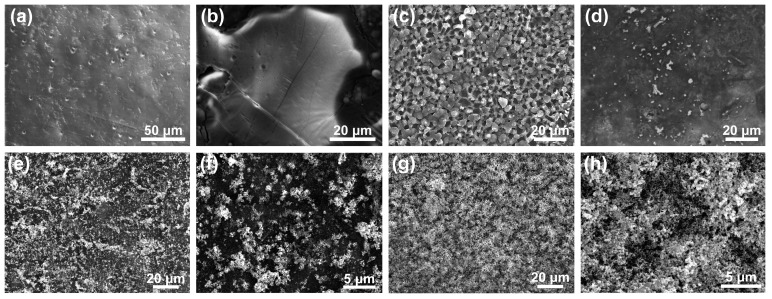

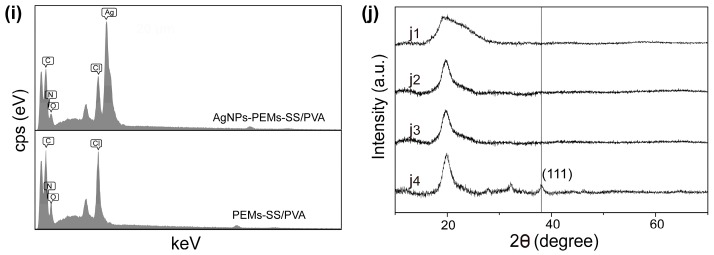
Surface morphologies of SS/PVA (**a**); PEMs-SS/PVA (**b**); cross-sectional structure of PEMs-SS/PVA films (**c**); SS/PVA film in silver nitrate solution irradiated by UV light for 10 min (**d**); PEMs-SS/PVA film in silver nitrate solution irradiated by UV light for 10 min (**e**,**f**) and 30 min (**g**,**h**). (**f**,**h**) are the high magnification of (**e**,**g**), respectively; EDS spectrum of PEMs-SS/PVA film and AgNPs-PEMs-SS/PVA film (**i**); XRD patterns of sericin (j1), SS/PVA film (j2), PEMs-SS/PVA film (j3) and AgNPs-PEMs-SS/PVA film (j4) (**j**).

SEM images were shown in Figure 2. The surface morphology of SS/PVA film was uniform, which showed good structural integrity without any cracks and interface layers (Figure 2a), indicating sericin and PVA has good compatibility. Figure 2b showed the film surface seemed to be covered by a layer of membranous-like substance after PEM coating on the surface of SS/PVA film. Figure 2c showed the cross-sectional structure of PEMs-SS/PVA film, suggesting PEMs formed a three-dimensional matrix. Figure 2d–f showed the morphologies of AgNPs-SS/PVA films without and with PEMs, respectively. High-density AgNPs were observed on the surface of PEMs-SS/PVA film, whereas only a small amount of AgNPs was on the surface of SS/PVA film without PEMs under the same reaction condition. The result indicated that PEM coating promoted the synthesis of high-density AgNPs. Previous studies indicate that the carboxyl groups of PAA greatly increase Ag^+^ adsorption and thus promote the transformation of Ag^+^ to Ag^0^ under UV irradiation [44]. With UV irradiation time increased to 30 min, AgNPs density on the surface of PEMs-SS/PVA film significantly increased (Figure 2g,h). The long-lasting antibacterial activity of AgNPs material is mainly dependent on the life of AgNPs. Hence, AgNPs-PEMs-SS/PVA film was expected to have durable antibacterial activity for its high density AgNPs.

The EDS spectrum showed the peaks of carbon, nitrogen, chlorine and oxygen element on PEMs-SS/PVA film (Figure 2i). The peaks of carbon, nitrogen, chlorine and oxygen were attributed to PAA, PDDA, PVA and sericin, respectively. The chlorine peak was mainly from PDDA. The EDS spectrum of AgNPs-PEMs-SS/PVA film showed a distinct peak of the silver element except carbon, nitrogen, chlorine, oxygen and chlorine peak.

XRD patterns were shown in Figure 2j. A broad peak located at 2θ = 19.2° was discovered in sericin pattern, which was consistent with the previous report [45]. The other three patterns had a characteristic broad peak located at 2θ = 19.8°, which could be assigned to the crystal structure of PVA. No significant change occurred on the pattern of SS/PVA film after PEM coating, UV irradiation and AgNPs modification, suggesting these treatments did not affect the crystal structure of SS/PVA film. After AgNPs modification, one peak appeared at 2θ = 38.2°, which could be ascribed to the crystal planes (111) of the face-centered cubic structure of AgNPs. Another peak appeared at 2θ = 32.2°, which could be attributed to the crystal planes (111) of Ag_2_O. UV irradiation facilitates the transformation of Ag^+^ to Ag^0^. At the same time, it will produce a small part of ozone. Ag_2_O may be derived from the oxidation of AgNPs on the film surface exposed to the UV-generated ozone [46,47] or air in aqueous solutions [48]. EDS and XRD results suggested that AgNPs were successfully synthesized and evenly distributed on the surface of PEMs-SS/PVA film. The high density and good crystallinity of AgNPs may greatly increase the antibacterial activity of PEMs-SS/PVA film.

### 2.3. FT-IR Analysis

**Figure 3:**
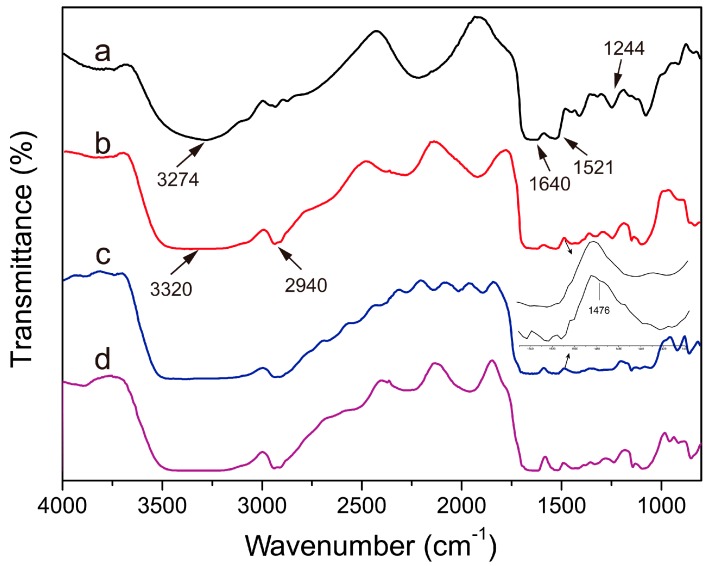
FT-IR spectra of sericin (**a**); SS/PVA (**b**); PEMs-SS/PVA (**c**) and AgNPs-PEMs-SS/PVA films (**d**).

FT-IR spectrum is a classical method to reveal the structure of a substance [49]. Figure 3 showed the FT-IR spectra of sericin (a), SS/PVA (b), PEMs-SS/PVA (c) and AgNPs-PEMs-SS/PVA films (d). Three characteristic peaks at 1640, 1521 and 1244 cm^−1^ were discovered in all samples, which may be assigned to C=O stretching vibration of amide I peak, a combination of C∓N stretching and N∓H bending vibration of amide II peak and a combination of N∓H bending and C∓N stretching vibration of amide III, respectively. Pure sericin and PVA have broad bands at 3274 cm^−1^ and 3340 cm^−1^, which are associated with the amine N∓H stretching vibration of sericin and the O∓H stretching vibration of PVA, respectively. After blending with PVA, the N∓H stretching vibration band of sericin slightly shifted from 3274 to 3320 cm^−1^, indicating that sericin and PVA had good compatibility. After PEM modification, a weak peak occurred at 1476 cm^−1^, which could be ascribed to the cycle vibration band of PDDA [50]. The results confirmed the presence of PEMs on the surface of SS/PVA film. No significant change was observed on the C∓O vibration band of AgNPs-PEMs-SS/PVA film. This may be because the carboxyl groups were involved in the adsorption of Ag^+^, but did not participate in the formation of AgNPs. The ∓CH_2_ characteristic peak of PVA at 2940 cm^−1^ may mask the ∓CH_3_ and ∓CH_2_ vibration band of PDDA at 2870 cm^−1^ and 2945 cm^−1^, respectively.

### 2.4. Wettability Measurement and Water Uptake Ability

**Figure 4:**
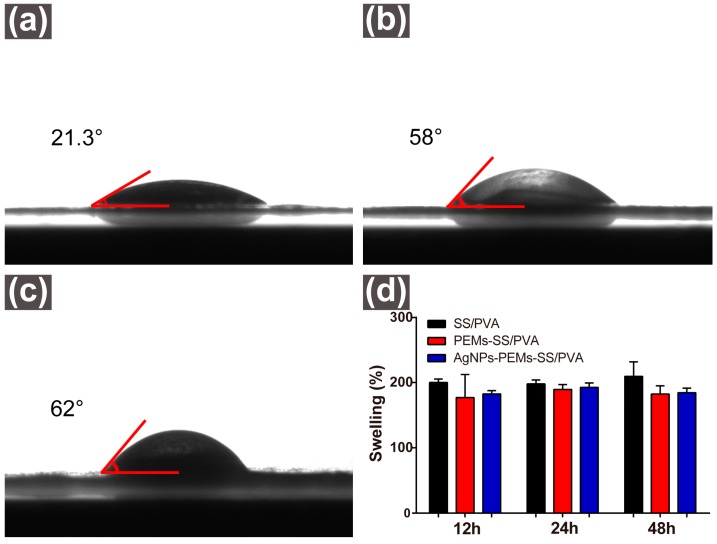
Water contact angles of SS/PVA (**a**); PEMs-SS/PVA (**b**); and AgNPs-PEMs-SS/PVA films (**c**); swelling ratios of SS/PVA, PEMs-SS/PVA and AgNPs-PEMs-SS/PVA films (**d**).

Figure 4a–c showed the wetting behavior of SS/PVA, PEMs-SS/PVA and AgNPs-PEMs-SS/PVA films assessed by water contact angle measurement, respectively. The water contact angle of SS/PVA film was 21.3°, indicating that SS/PVA film had excellent hydrophilicity. After PEM coating, the water contact angle of SS/PVA film increased to 58°, indicating its good hydrophilicity. PEM coating on SS/PVA film may reduce the porosity of SS/PVA film, thus resulting in the increase of water contact angle of SS/PVA film. In addition, time delay from making the film to water contact angle measurement could result in surface saturation with hydrocarbons and thus cause the increase of water contact angle. AgNPs-PEMs-SS/PVA film had a water contact angle of 62°. The water contact angle was hardly changed before and after AgNPs modification, indicating that AgNPs modification did not affect the hydrophilicity of PEMs-SS/PVA film. Karumuri et al. have reported that water contact angle has slightly gone down with AgNPs deposition, but the small difference does not affect the hydrophilicity [51].

The swelling properties of SS/PVA, PEMs-SS/PVA and AgNPs-PEMs-SS/PVA films were examined (Figure 4d). All samples (1 × 1 cm^2^) were immersed in PBS buffer (pH 7.4) for 12 h, 24 h and 48 h, respectively. The swelling ratios of these films were about 1.8 without significant differences, indicating these films had good hygroscopicity. The swelling properties were not significantly different after 12 h, 24 h and 48 h because the total accessible surface of these films had been completely saturated by water molecules quickly. These results indicated that SS/PVA, PEMs-SS/PVA and AgNPs-PEMs-SS/PVA films had good hydrophilicity and water absorption capability.

### 2.5. Mechanical Property

**Figure 5:**
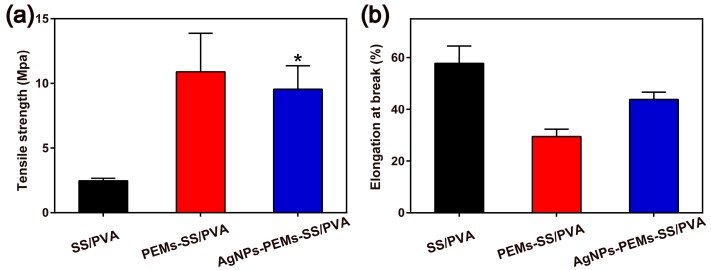
Mechanical properties of SS/PVA, PEMs-SS/PVA and AgNPs-PEMs-SS/PVA films: (**a**) tensile strength and (**b**) elongation at break. Data are the average value plus standard deviation (SD) from three independent experiments. Statistical significance is assessed using an unpaired *t*-test. * indicates *p* < 0.05.

The tensile strength and elongation at break of these films were measured, as shown in Figure 5. SS/PVA film had a tensile strength of 2.45 MPa, which was much lower than that of the modified films. PEM coating and AgNPs modification resulted in the increase of the tensile strength about three times compared with that of SS/PVA film, suggesting that PEM coating and AgNPs modification increased the stiffness of SS/PVA film. It may be attributed to the increase of film thickness. Elongation at break represents the flexibility of material [52]. Although the elongation at break (strain) of AgNPs-PEMs-SS/PVA film had gone down a little gone compared with that of SS/PVA film, the change was not statistically different. The strain of AgNPs-PEMs-SS/PVA film was 43.80 ± 3.2%, which still could support its application as an antibacterial biomaterial. PEM coating and AgNPs modification increased the film thickness, thus resulting in a slight decrease of the film flexibility. In general, the mechanical performance of AgNPs-PEMs-SS/PVA film was able to support its application as an antibacterial biomaterial.

### 2.6. TGA Analysis

**Figure 6:**
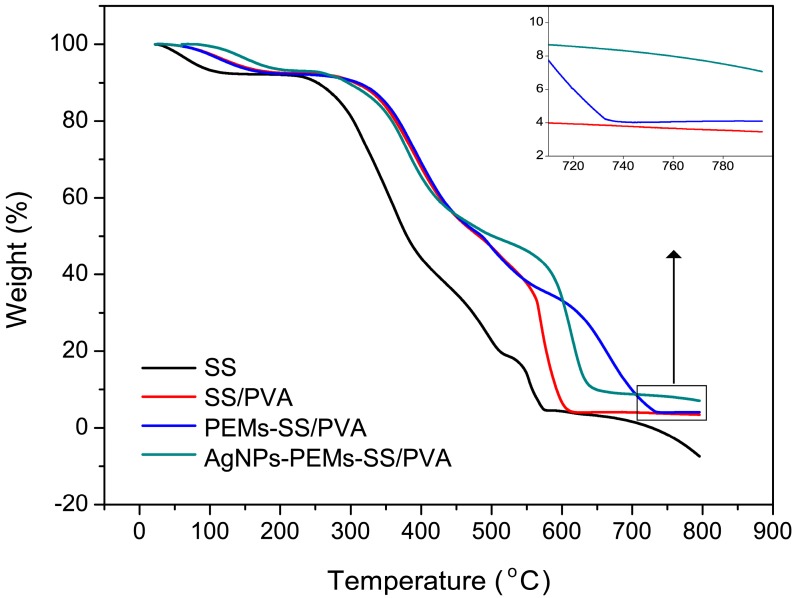
TGA curves of sericin, SS/PVA, PEMs-SS/PVA and AgNPs-PEMs-SS/PVA films.

TGA reflects that the mechanism led to a unique thermal behavior. The result showed SS/PVA film underwent a multi-steps degradation process (Figure 6). In 0–100 °C, the initial weight loss was due to the presence of moisture. While increasing temperature to 200 °C, the observed weight loss may be attributed to the degradation of the exposed side chains and the breakdown of main chain groups of sericin. After blending with PVA, water evaporation caused the reduction of initial weight from room temperature to 180 °C. At this stage, the weight loss of SS/PVA film was slower than that of sericin, which may be due to the fact that SS/PVA film contained more bound water than sericin itself did. The second stage of weight loss occurred from 200 to 600 °C. This may be ascribed to the degradation of the exposed side chains and the breakdown of main chain groups of sericin and PVA. The weight loss of SS/PVA film reached an equilibrium at around 620 °C, which was higher than that of sericin. SS/PVA film had about 3% residual weight, suggesting that the hydrogen-bond interaction between sericin and PVA may be favorable to the thermal stability of SS/PVA blend film. After PEM coating, the equilibrium temperature increased to around 730 °C. PEMs-SS/PVA film had about 4% residual weight, indicating that PEM coating could enhance the thermostability of SS/PVA film and delay its thermal degradation. After AgNPs modification, the residual weight increased to about 7%, and the equilibrium temperature increased to around 650 °C, implying that AgNPs may improve the thermostability of PEMs-SS/PVA film and reduce the weight loss. The result also indicated that PEMs and AgNPs were successfully modified on the surface of SS/PVA film.

### 2.7. Inhibition Zone Assay

**Figure 7:**
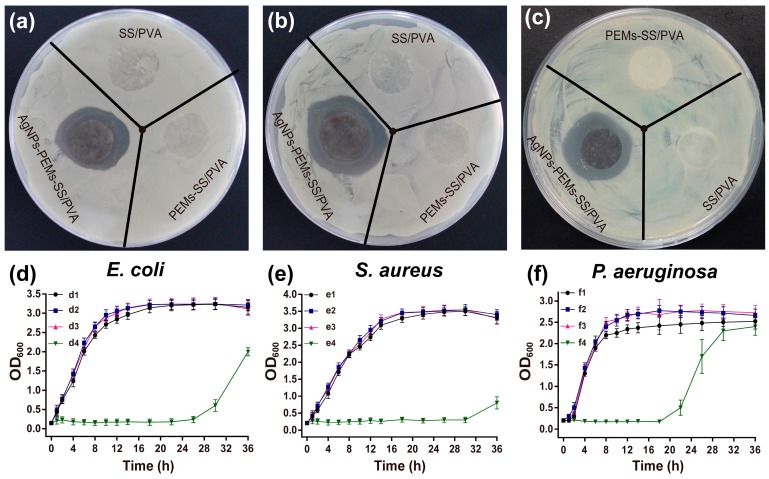
Antibacterial effects of SS/PVA, PEMs-SS/PVA, AgNPs-PEMs-SS/PVA films against *E. coli* (**a**); *S. aureus* (**b**) and *P. aeruginosa* (**c**); Growth curves assays of *E. coli* (**d**); *S. aureus* (**e**) and *P. aeruginosa* (**f**). Bacteria without treatment (d1, e1, f1); Bacteria in the presence of SS/PVA (d2, e2, f2), PEMs-SS/PVA (d3, e3, f3) and AgNPs-PEMs-SS/PVA films (d4, e4, f4).

The antibacterial effects of AgNPs-PEMs-SS/PVA films were assessed against Gram-negative bacteria (*E. coli* and *P. aeruginosa*) and Gram-positive bacteria (*S. aureus*), respectively (Figure 7). SS/PVA and PEMs-SS/PVA films did not exhibit significant bactericidal effects against either *E. coli*, *P. aeruginosa* or *S. aureus*. However, after AgNPs modification, the film could effectively kill bacteria and form evident inhibition zones with an approximate average diameter of 2.5 cm. The diameters of the inhibition zones were summarized in Table 1. The result suggested that AgNPs-PEMs-SS/PVA film obviously inhibited the growth of *E. coli* (a), *S. aureus* (b) and *P. aeruginosa* (c).

### 2.8. Bacterial Growth Curve Assay

Bacterial growth curve experiments were carried out to further evaluate the antibacterial effects of AgNPs-PEMs-SS/PVA films. The optical density of bacteria at 600 nm (OD_600_) was used to evaluate bacterial growth in the presence of the films. As shown in Figure 7d–f, the lag phase of *E. coli*, *S. aureus* and *P. aeruginosa* was less than 1 h in the presence of SS/PVA and PEMs-SS/PVA films. However, in the presence of AgNPs-PEMs-SS/PVA films, the lag phase of *E. coli*, *P. aeruginosa* and *S. aureus* significantly prolonged to 26 h, 22 h and 30 h, respectively. For AgNPs film without PEMs, the lag phase of bacteria generally prolongs to 12 h [8]. Compared to AgNPs film without PEMs, AgNPs-PEMs-SS/PVA film had a more significant inhibitory effect on the bacterial growth. The results demonstrated that AgNPs-PEMs-SS/PVA film had excellent antimicrobial activity.

### 2.9. Antimicrobial Stability Analysis

**Figure 8:**
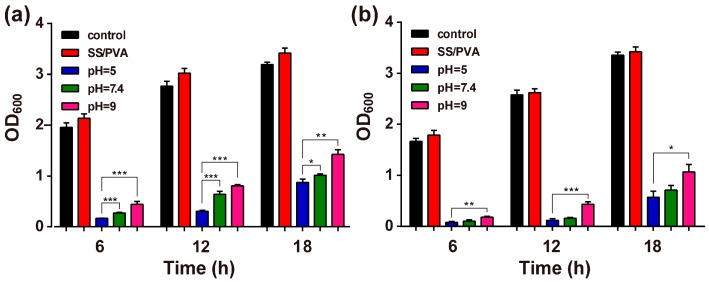
Durable bactericidal activity of AgNPs-PEMs-SS/PVA films against *E. coli* (**a**) and *S. aureus* (**b**). Data are the average value plus standard deviation (SD) from three independent experiments. Statistical significance is assessed using an unpaired *t*-test. * Indicates *p* < 0.05; ** indicates *p* < 0.01; *** indicates *p* < 0.001.

Figure 8a,b showed the antimicrobial stability analysis of AgNPs-PEMs-SS/PVA films against *E. coli* and *S. aureus*, after the films were treated in different pHs (5, 7.4, 9) for 24 h. OD_600_ was used to evaluate bacterial growth in the presence of the treated AgNPs-PEMs-SS/PVA films. After 18 h, the film still showed considerable antimicrobial activity compared with that of the control. The antimicrobial activity of the film treated under pH 5 condition was higher than that of the film treated under pH 7.4 or pH 9 conditions. This may be due to the fact that AgNPs-PEMs-SS/PVA film was more stable and AgNPs loss was less under pH 5 condition than that under other conditions. The result indicated that AgNPs-PEMs-SS/PVA film had durable and steady antibacterial activity.

### 2.10. Mass Loss Analysis

**Figure 9:**
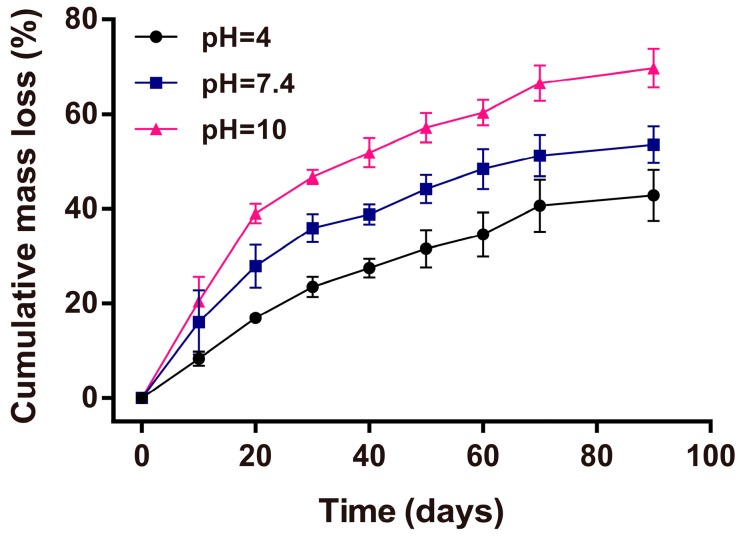
Mass loss analysis of AgNPs-PEMs-SS/PVA films under pH 4–10 conditions.

The stability of antibacterial reagent is crucial for the performance of a wound dressing material. As shown in Figure 9, about 40% and 50% mass loss of AgNPs-PEMs-SS/PVA films occurred after being treated for 90 days under pH 4 and pH 7.4 conditions, respectively. However, under the pH 10 condition, about 70% mass loss occurred, which was faster than that under pH 4 and pH 7.4 conditions. Sericin has 24% of acidic amino acids (Glu and Asp) and 8% of alkaline amino acids (Lys, His and Arg). The content of acidic amino acids is higher than that of alkaline amino acids. Thus, sericin has an isoelectric point of approximately 3.8 [53]. Moreover, PVA shows weak acidity in water. Hence, the SS/PVA blend film and its degraded products are expected to be slightly acidic. The result indicated that the mass loss of AgNPs-PEMs-SS/PVA film under pH 10 condition was faster than that under pH 4 and pH 7.4 conditions. It was reasonable as an alkaline condition could be neutralized by the acidity of sericin and PVA, thus promoting the degradation and mass loss of SS/PVA film. As we know, pure sericin film is not stable in solution as it will be degraded in a short time. Overall, the film still kept good integrity with quite a certain percentage of residual mass after long-term storage, indicating that it had good stability. The stability may be ascribed to the protective effect of PEMs and AgNPs on SS/PVA film. The result suggested that AgNPs-PEMs-SS/PVA film had good stability under different pH conditions, which had potential value as an antibacterial material. In addition, the film lost its mass slowly in pH 4–10 buffers, suggesting that the film was degradable.

## 3. Materials and Methods

### 3.1. Materials

Silkworm cocoons were supplied by the State Key Laboratory of Silkworm Genome Biology, Southwest University (Chongqing, China). PVA, PEI (50% in water, MW 750,000 Da), PAA (MW 3000 Da), PDDA (MW 200,000–350,000 Da) and silver nitrate (AgNO_3_) (AR, 99.99%) were products from Aladdin Corp. (Shanghai, China). MiliQ water was provided by a MilliQ water purification system from Millipore Corp. (Billerica, MA, USA) and used in the experiment.

### 3.2. Preparation of PEMs-SS/PVA Film

Sericin was extracted according to a previous report with minor modification [54]. PVA was dissolved in water under a constant stirring speed at 80 °C until it was fully dissolved to a final concentration of 5% (*w/t*). Sericin solution (4%, *w/t*) and PVA solution (5%, *w/t*) were mixed together with a 1:1 ratio at 60 °C for 30 min at least. Then, the mixed solution was frozen under −20 °C and thawed at room temperature for four cycles to form SS/PVA hydrogel. After drying at room temperature, the hydrogel became SS/PVA blend film. First, SS/PVA film was immersed in PEI solution (2%, *w/v*) for 2 min to functionalize the surface with positive charges. After washing with water, the blend film was treated with PAA solution (2%, *w/v*) for 2 min to adsorb a layer of negative charges. The opposite charges interaction between PAA and PEI facilitated the binding of PAA to the surface of the film. Thereafter, the blend film was immersed into PDDA solution (2%, *w/v*) for 2 min to functionalize the surface of SS/PVA film with a layer of positive charges. Finally, the blend film was immobilized with a layer of negative charges by immersing into PAA solution (2%, *w/v*) for 2 min.

### 3.3. Synthesis of AgNPs In Situ on PEMs-SS/PVA Film

UV-assisted AgNPs synthesis has been carried out to prepare AgNPs in situ on sericin hydrogel [55]. In this work, PEMs-SS/PVA film was soaked in 20 mM AgNO_3_ solution and then irradiated with 365 nm UV light (3 W) for 10–30 min to synthesize AgNPs in situ on the film at room temperature. The distance between UV light and PEM-SS/PVA film was 8 cm. The solution temperature during UV irradiation was 25 °C. After drying at room temperature, AgNPs-PEMs-SS/PVA film was collected for the following experiment.

### 3.4. Materials Characterization

The morphologies of SS/PVA, PEMs-SS/PVA and AgNPs-PEMs-SS/PVA films were imaged on a JEOL SEM of JCM-5000 (Tokyo, Japan) after platinum plating. During SEM test, EDS spectra were collected on an INCA X-Max 250 (Oxford, UK) to analyze chemical elements of the samples. AgNPs modified and unmodified SS/PVA films were examined using XRD on a PANalytical X-ray diffractometer of X’Pert Powder with a 2θ range of 10–80° (Almelo, Netherlands). FT-IR measurements were performed in the wavenumber of 4000–800 cm^−1^ during 64 scans with 2 cm^−1^ resolution on a Thermo-Fisher Scientific FT-IR spectrometer of Nicolet iz10 (Waltham, MA, USA). TGA was carried out to evaluate the thermal stability of the film on a TA Instruments analyzer of TGA-Q50 (New Castle, DE, USA) under an air flow at a heating rate of 10 °C/min from room temperature to 800 °C. In a desired temperature range, if the film is thermally stable, no mass change will be observed. Negligible mass loss corresponds to little or no slope in the TGA trace. TGA also gives the upper temperature limitation of the film. Beyond this temperature, the film will degrade.

### 3.5. Wettability Measurement

The wettability of SS/PVA, PEMs-SS/PVA and AgNPs-PEMs-SS/PVA films were measured at room temperature via sessile drop contact angle measurement on a Krüss DSA100 contact angle system (Hamburg, Germany). At five different positions, a water droplet of 4 μL was dropped on the surface of the film. The contact angle was measured and determined as the average value of five measurements.

### 3.6. Swelling Properties

Swelling property was studied according to Mandal et al. [56]. The dried samples were weighed as *W*_1_ in a dry condition and soaked into 10 mL phosphate buffer (PBS, pH 7.4) at 37 °C. At various time intervals, the tested films were removed from PBS buffer and excess water was wiped off from the film surface, and then immediately weighed as *W*_2_. Each sample was repeated at least three times under the same condition. The average value was used to evaluate the swellability of the film. Swelling ratio (*S*) was calculated according to the following equation:

*S* (%) = (*W*_2_ − *W*_1_) × 100%/*W*_1_

### 3.7. Mechanical Analysis

Mechanical analysis was measured at a constant temperature and humidity condition on a Shimadzu universal testing machine of AG-Xplus (Kyoto, Japan) equipped with a 1000-N load cell at a crosshead speed of 3 mm/min. The samples were cut into pieces with dimensions of 4 × 1 cm^2^ (length × width). The thickness of each sample was determined by SEM. The original data were transformed into true stress (σ) and strain (ε) to plot the stress–strain curves [57].

### 3.8. Inhibition Zone Assay

Inhibition zone test was carried out according to Schillinger and Lucke [58]. *E. coli*, *S. aureus* and *P. aeruginosa* were inoculated into 100 mL Luria-Bertani medium (pH 7.4) at 37 °C under a constant shaking speed of 220 rpm. While the OD_600_ of bacteria reached 1.0, bacterial suspentions (200 μL) were spread on agar medium plates in the presence of circular AgNPs modified or unmodified SS/PVA films (*d* = 1.50 cm). After incubation at 37 °C overnight, the antibacterial activities of the samples were evaluated according to the diameters of the formed bacterial inhibition zones.

### 3.9. Growth Curve Analysis

Bacterial growth curve assay was conducted according to Pal’s protocol [59] with a slight modification. Bacteria at lag phase were inoculated into 10 mL LB medium (pH 7.4) in the presence of AgNPs modified or unmodified SS/PVA films (1 × 1 cm^2^), and cultured with a constant shaking speed of 220 rpm at 37 °C. About 0.5 mL bacterial suspensions were collected at different intervals to measure OD_600_. All tests were performed in triplicate to ensure the reproducibility of the experiment. The average value of three independent measurements was used to evaluate the bacterial growth in the presence of the film.

### 3.10. Antimicrobial Stability Test

To analyze the antimicrobial stability of AgNPs-PEMs-SS/PVA film, the films were cut into small pieces with dimensions of 1 × 1 cm^2^ (length × width), then soaked in PBS buffers (pH 5, 7.4 and 9) for 24 h, respectively. Then, the films were taken out from buffers, washed and dried. Next, bacteria were cultured in the presence of the treated AgNPs-PEMs-SS/PVA films. At various time intervals, bacteria were collected and OD_600_ was measured to evaluate the antimicrobial activity of the treated films.

### 3.11. Mass Loss Analysis

AgNPs-PEMs-SS/PVA films were cut into small pieces with dimensions of 3 cm × 3 cm (length × width) and immersed in PBS buffers (pH 4, 7.4, 10) for 90 days at 37 °C. PBS buffer was replaced daily to keep fresh. The initial dry mass of the film was weighed as *W_0_*. At given time points, the films were taken out from PBS buffers, washed, dried, and weighed as *W_t_*. Mass loss was calculated as the following equation:

*M_L_* (%) = (*W*_0_ − *W_t_*) × 100%/*W*_0_

All tests were performed in triplicate and the average value was used to assess the mass loss of the films.

## 4. Conclusions

In this study, we have developed a simple, economic and green method to prepare AgNPs-PEMs-SS/PVA film via a layer-by-layer self-assembly technique and UV-assisted AgNPs synthesis method. The synthesized AgNPs have good crystal structures. PEMs serve as a 3D matrix for the high density growth of AgNPs and the protection layer to prevent AgNPs from oxidation and falling off, thus endowing SS/PVA film with highly effective and durable antibacterial activity. The prepared AgNPs-PEMs-SS/PVA film has good mechanical performance, hydrophilicity, water absorption capability as well as excellent and durable antibacterial activity and good stability and degradability. This novel film is expected to be applied in antibacterial biomaterials.

## Figures and Tables

**Table 1:** Diameters of inhibition zones of SS/PVA, PEMs-SS/PVA and AgNPs-PEMs-SS/PVA films against *E. coli*, *S. aureus* and *P. aeruginosa*.

Bacteria	Control (cm)	PEMs-SS/PVA (cm)	AgNPs-PEMs-SS/PVA (cm)
*E. coil*	1.55 ± 0.02	1.55 ± 0.03	2.17 ± 0.42
*P. aeruginosa*	1.55 ± 0.01	1.55 ± 0.02	2.49 ± 0.41
*S. aureus*	1.55 ± 0.03	1.55 ± 0.04	2.82 ± 0.26

## References

[1] Zhang Y.Q., Ma Y., Xia Y.Y., Shen W.D., Mao J.P., Xue R.Y. (2006). Silk sericin-insulin bioconjugates: Synthesis, characterization and biological activity. J. Control. Release.

[2] Padamwar M.N., Pawar A.P. (2004). Silk sericin and its applications: A review. J. Sci. Ind. Res. India.

[3] Ude A.U., Eshkoor R.A., Zulkifili R., Ariffin A.K., Dzuraidah A.W., Azhari C.H. (2014). *Bombyx mori* silk fibre and its composite: A review of contemporary developments. Mater. Des..

[4] Rajasekaran E., Jency S., Panneerselvam K. (2011). Carbon profile of commercially important sericin proteins of silkworm, *bombyx mori*. J. Adv. Bioinform. Appl. Res. ISSN.

[5] Aramwit P., Sangcakul A. (2007). The effects of sericin cream on wound healing in rats. Biosci. Biotechnol. Biochem..

[6] Aramwit P., Kanokpanont S., Nakpheng T., Srichana T. (2010). The effect of sericin from various extraction methods on cell viability and collagen production. Int. J. Mol. Sci..

[7] Kundu S.C., Dash B.C., Dash R., Kaplan D.L. (2008). Natural protective glue protein, sericin bioengineered by silkworms: Potential for biomedical and biotechnological applications. Prog. Polym. Sci..

[8] He H., Cai R., Wang Y., Tao G., Guo P., Zuo H., Chen L., Liu X., Zhao P., Xia Q. (2017). Preparation and characterization of silk sericin/pva blend film with silver nanoparticles for potential antimicrobial application. Int. J. Biol. Macromol..

[9] Lewis K., Klibanov A.M. (2005). Surpassing nature: Rational design of sterile-surface materials. Trends Biotechnol..

[10] Saeki D., Nagao S., Sawada I., Ohmukai Y., Maruyama T., Matsuyama H. (2013). Development of antibacterial polyamide reverse osmosis membrane modified with a covalently immobilized enzyme. J. Membr. Sci..

[11] Ben-Sasson M., Zodrow K.R., Qi G.G., Kang Y., Giannelis E.P., Elimelech M. (2014). Surface functionalization of thin-film composite membranes with copper nanoparticles for antimicrobial surface properties. Environ. Sci. Technol..

[12] Cai R., Tao G., He H., Song K., Zuo H., Jiang W., Wang Y. (2017). One-step synthesis of silver nanoparticles on polydopamine-coated sericin/polyvinyl alcohol composite films for potential antimicrobial applications. Molecules.

[13] Nel A., Xia T., Madler L., Li N. (2006). Toxic potential of materials at the nanolevel. Science.

[14] Moore M.N. (2006). Do nanoparticles present ecotoxicological risks for the health of the aquatic environment?. Environ. Int..

[15] Grätzel M. (2001). Photoelectrochemical cells. Nature.

[16] Shiraishi Y., Toshima N. (1999). Colloidal silver catalysts for oxidation of ethylene. J. Mol. Catal. A-Chem..

[17] White R.J., Budarin V.L., Moir J.W.B., Clark J.H. (2011). A sweet killer: Mesoporous polysaccharide confined silver nanoparticles for antibacterial applications. Int. J. Mol. Sci..

[18] Groneberg D.A., Giersig M., Welte T., Pison U. (2006). Nanoparticle-based diagnosis and therapy. Curr. Drug Targets.

[19] Lara H.H., Garza-Trevino E.N., Ixtepan-Turrent L., Singh D.K. (2011). Silver nanoparticles are broad-spectrum bactericidal and virucidal compounds. J. Nanobiotechnol..

[20] Tao G., Wang Y.J., Liu L.N., Chang H.P., Zhao P., He H.W. (2016). Preparation and characterization of silver nanoparticles composited on polyelectrolyte film coated sericin gel for enhanced antibacterial application. Sci. Adv. Mater..

[21] Tao G., Cai R., Wang Y., Song K., Guo P., Zhao P., Zuo H., He H. (2017). Biosynthesis and characterization of AgNPs–silk/PVA film for potential packaging application. Materials.

[22] Dorjnamjin D., Ariunaa M., Shim Y.K. (2008). Synthesis of silver nanoparticles using hydroxyl functionalized ionic liquids and their antimicrobial activity. Int. J. Mol. Sci..

[23] Morones J.R., Elechiguerra J.L., Camacho A., Holt K., Kouri J.B., Ramirez J.T., Yacaman M.J. (2005). The bactericidal effect of silver nanoparticles. Nanotechnology.

[24] Skladanowski M., Golinska P., Rudnicka K., Dahm H., Rai M. (2016). Evaluation of cytotoxicity, immune compatibility and antibacterial activity of biogenic silver nanoparticles. Med. Microbiol. Immunol..

[25] Elliott C. (2010). The effects of silver dressings on chronic and burns wound healing. Br. J. Nurs..

[26] Darroudi M., Ahmad M.B., Zak A.K., Zamiri R., Hakimi M. (2011). Fabrication and characterization of gelatin stabilized silver nanoparticles under UV-light. Int. J. Mol. Sci..

[27] Kilin D.S., Prezhdo O.V., Xia Y.N. (2008). Shape-controlled synthesis of silver nanoparticles: Ab *initio* study of preferential surface coordination with citric acid. Chem. Phys. Lett..

[28] Wang M., Fu J., Huang D., Zhang C., Xu Q. (2013). Silver nanoparticles-decorated polyphosphazene nanotubes: Synthesis and applications. Nanoscale.

[29] Bin Ahmad M., Lim J.J., Shameli K., Ibrahim N.A., Tay M.Y. (2011). Synthesis of silver nanoparticles in chitosan, gelatin and chitosan/gelatin bionanocomposites by a chemical reducing agent and their characterization. Molecules.

[30] Cintas P., Palmisano G., Cravotto G. (2011). Power ultrasound in metal-assisted synthesis: From classical barbier-like reactions to click chemistry. Ultrason. Sonochem..

[31] Chang S.Q., Kang B., Dai Y.D., Chen D. (2009). Synthesis of antimicrobial silver nanoparticles on silk fibers via gamma-radiation. J. Appl. Polym. Sci..

[32] Wang X.M., Gao W.R., Xu S.P., Xu W.Q. (2012). Luminescent fibers: In situ synthesis of silver nanoclusters on silk via ultraviolet light-induced reduction and their antibacterial activity. Chem. Eng. J..

[33] Wei J.J., Wang L.M., Zhang X., Ma X.J., Wang H., Su Z.H. (2013). Coarsening of silver nanoparticles in polyelectrolyte multilayers. Langmuir.

[34] Hu R., Li G.Z., Jiang Y.J., Zhang Y., Zou J.J., Wang L., Zhang X.W. (2013). Silver-zwitterion organic-inorganic nanocomposite with antimicrobial and antiadhesive capabilities. Langmuir.

[35] Li Q.L., Mahendra S., Lyon D.Y., Brunet L., Liga M.V., Li D., Alvarez P.J.J. (2008). Antimicrobial nanomaterials for water disinfection and microbial control: Potential applications and implications. Water Res..

[36] Mollahosseini A., Rahimpour A., Jahamshahi M., Peyravi M., Khavarpour M. (2012). The effect of silver nanoparticle size on performance and antibacteriality of polysulfone ultrafiltration membrane. Desalination.

[37] Decher G. (1997). Fuzzy nanoassemblies: Toward layered polymeric multicomposites. Science.

[38] Ariga K., Hill J.P., Ji Q.M. (2007). Layer-by-layer assembly as a versatile bottom-up nanofabrication technique for exploratory research and realistic application. Phys. Chem. Chem. Phys..

[39] Wang H.S., Qiao X.L., Chen J.G., Wang X.J., Ding S.Y. (2005). Mechanisms of PVP in the preparation of silver nanoparticles. Mater. Chem. Phys..

[40] Zhang D.S., Toh G.W., Lin H., Chen Y.Y. (2012). In situ synthesis of silver nanoparticles on silk fabric with PNP for antibacterial finishing. J. Mater. Sci..

[41] Xiang Y., Lu S.F., Jiang S.P. (2012). Layer-by-layer self-assembly in the development of electrochemical energy conversion and storage devices from fuel cells to supercapacitors. Chem. Soc. Rev..

[42] Dubas S.T., Kumlangdudsana P., Potiyaraj P. (2006). Layer-by-layer deposition of antimicrobial silver nanoparticles on textile fibers. Colloid Surf. A.

[43] Ito K., Saito A., Fujie T., Miyazaki H., Kinoshita M., Saitoh D., Ohtsubo S., Takeoka S. (2016). Development of a ubiquitously transferrable silver-nanoparticle-loaded polymer nanosheet as an antimicrobial coating. J. Biomed. Mater. Res. B.

[44] Machado G., Beppu M.M., Feil A.F., Figueroa C.A., Correia R.R.B., Teixeira S.R. (2009). Silver nanoparticles obtained in PAH/PAA-based multilayers by photochemical reaction. J. Phys. Chem. C.

[45] Tao W., Li M.Z., Xie R.J. (2005). Preparation and structure of porous silk sericin materials. Macromol. Mater. Eng..

[46] Waterhouse G., Bowmaker G., Metson J. (2001). Oxidation of a polycrystalline silver foil by reaction with ozone. Appl. Surf. Sci..

[47] Xiong Y.Q., Liu L.M., Lu W.G., Yang D.Q., Da D.A. (2001). Atomic force microscopy and x-ray photoelectron spectroscopy study on nanostructured silver thin films irradiated by atomic oxygen. Mater. Sci. Eng. B.

[48] Liu J., Hurt R.H. (2010). Ion release kinetics and particle persistence in aqueous nano-silver colloids. Environ. Sci. Technol..

[49] Ki C.S., Kim J.W., Oh H.J., Lee K.H., Park Y.H. (2007). The effect of residual silk sericin on the structure and mechanical property of regenerated silk filament. Int. J. Biol. Macromol..

[50] Liu S.N., Cai C.X. (2007). Immobilization and characterization of alcohol dehydrogenase on single-walled carbon nanotubes and its application in sensing ethanol. J. Electroanal. Chem..

[51] Karumuri A.K., Oswal D.P., Hostetler H.A., Mukhopadhyay S.M. (2016). Silver nanoparticles supported on carbon nanotube carpets: Influence of surface functionalization. Nanotechnology.

[52] Kumar P.T.S., Lakshmanan V.K., Anilkumar T.V., Ramya C., Reshmi P., Unnikrishnan A.G., Nair S.V., Jayakumar R. (2012). Flexible and microporous chitosan hydrogel/nano zno composite bandages for wound dressing: *In vitro* and *in vivo* evaluation. ACS Appl. Mater. Interfaces.

[53] Wang Z., Zhang Y.S., Zhang J.X., Huang L., Liu J., Li Y.K., Zhang G.Z., Kundu S.C., Wang L. (2014). Exploring natural silk protein sericin for regenerative medicine: An injectable, photoluminescent, cell-adhesive 3d hydrogel. Sci. Rep.-UK.

[54] Wu J.H., Wang Z., Xu S.Y. (2007). Preparation and characterization of sericin powder extracted from silk industry wastewater. Food Chem..

[55] Tao G., Liu L.N., Wang Y.J., Chang H.P., Zhao P., Zuo H., He H.W. (2016). Characterization of silver nanoparticle in situ synthesis on porous sericin gel for antibacterial application. J. Nanomater..

[56] Vazquez B., Roman J.S., Peniche C., Cohen M.E. (1997). Polymeric hydrophilic hydrogels with flexible hydrophobic chains. Control of the hydration and interactions with water molecules. Macromolecules.

[57] Guan J., Porter D., Vollrath F. (2013). Thermally induced changes in dynamic mechanical properties of native silks. Biomacromolecules.

[58] Schillinger U., Lucke F.K. (1989). Antibacterial activity of lactobacillus-sake isolated from meat. Appl. Environ. Microbiol..

[59] Pal S., Tak Y.K., Song J.M. (2007). Does the antibacterial activity of silver nanoparticles depend on the shape of the nanoparticle? A study of the gram-negative bacterium *escherichia coli*. Appl. Environ. Microbiol..

